# A national assessment of urban forest carbon storage and sequestration in Canada

**DOI:** 10.1186/s13021-023-00230-4

**Published:** 2023-07-08

**Authors:** James W. N. Steenberg, Melissa Ristow, Peter N. Duinker, Lyna Lapointe-Elmrabti, J. Douglas MacDonald, David J. Nowak, Jon Pasher, Corey Flemming, Cameron Samson

**Affiliations:** 1 School for Resource and Environmental Studies, Dalhousie University, 6100 University Avenue, Halifax, NS B3H 4R2 Canada; 2 Environment and Climate Change Canada, Gatineau, Canada; 3 USDA Forest Service, Northern Research Station, Syracuse, USA

**Keywords:** Canopy cover, Greenhouse gases (GHG), Climate change, Mitigation, National inventory, Land sector

## Abstract

During a time of rapid urban growth and development, it is becoming ever more important to monitor the carbon fluxes of our cities. Unlike Canada’s commercially managed forests that have a long history of inventory and modelling tools, there is both a lack of coordinated data and considerable uncertainty on assessment procedures for urban forest carbon. Nonetheless, independent studies have been carried out across Canada. To improve upon Canada’s federal government reporting on carbon storage and sequestration by urban forests, this study builds on existing data to develop an updated assessment of carbon storage and sequestration for Canada’s urban forests. Using canopy cover estimates derived from ortho-imagery and satellite imagery ranging from 2008 to 2012 and field-based urban forest inventory and assessment data from 16 Canadian cities and one US city, this study found that Canadian urban forests store approximately 27,297.8 kt C (− 37%, + 45%) in above and belowground biomass and sequester approximately 1497.7 kt C year^−1^ (− 26%, + 28%). In comparison with the previous national assessment of urban forest carbon, this study suggested that in urban areas carbon storage has been overestimated and carbon sequestration has been underestimated. Maximizing urban forest carbon sinks will contribute to Canada’s mitigation efforts and, while being a smaller carbon sink compared to commercial forests, will also provide important ecosystem services and co-benefits to approximately 83% of Canadian people.

## Introduction

Climate change is a global problem and the need for substantive mitigation actions is immediate if warming is to be kept to 1.5 ℃ or well below 2 ℃, as outlined in the Paris Agreement. The land sector (e.g., forestry, agriculture, human settlements) is an important part of the natural carbon cycle and the greenhouse gas (GHG) emissions and removals associated with human management of the land sector carbon sinks are a key component of mitigation efforts. While major forested biomes like the boreal and tropical rainforest have the greatest capacity to affect the global climate, it is important that policies and management efforts aim to maximize forest sinks in all settings and land uses, including urban forests that are directly managed or modified by human activity and can perform as nature-based solutions to climate change. In this study, urban forest refers to all trees within an urban area, regardless of land use or degree of naturalization. Any urban tree contributing to canopy cover, whether it be a commercial street tree, residential tree, or forested patch within an urban area, is part of the urban forest.

During a time of rapid urban growth and development, it is becoming ever more important to monitor the carbon fluxes of our cities [14]. Urban trees make up an important part of standing woody biomass in Canada that is under direct, deliberate, and intensive management by humans. Maintaining and building larger and healthier urban forests will provide an opportunity for increasing the removal of atmospheric carbon while also providing a host of other co-benefits for urban populations, including improving human health, reducing pollution, and improving infrastructure longevity [4, 24, 31]. In addition to the climate change mitigation benefits associated with trees as carbon sinks, urban forests have the ability to provide carbon benefits indirectly by reducing the urban heat island effect and improving energy-use efficiency in buildings [4, 13, 20]. Effectively capturing and reporting on trends in urban trees helps document the overall trend in carbon in the land sector.

Nowak et al. [19] developed an assessment approach for the United States for carbon uptake by urban forests. The Nowak et al. [19] analysis used aerial imagery to calculate urban forest canopy cover and estimated total carbon storage and sequestration for urban centres across the US using the i-Tree Eco model, which is a software package developed by the USDA Forest Service [29]. The i-Tree Eco model calculates carbon storage and sequestration values, among other structural attributes and ecosystem services, for urban forests using urban sample plots or complete tree inventories [18]. For US urban forests, the gross sequestration rate value was estimated at 2.77 t C ha^−1^ year^−1^ (standard error = 0.45 t C ha^−1^ year^−1^) of canopy cover and storage was estimated at 76.9 t C ha^−1^ (standard error = 13.6 t C ha^−1^) of canopy cover. Pasher et al. [22] followed a similar approach for estimating carbon storage and sequestration in Canadian urban forests; however the study itself relied solely upon US data. The Nowak et al. [19] values for US carbon storage rates were changed to a gross sequestration rate of 2.12 t C ha^−1^ year^−1^, to reflect Canada’s shorter growing season.

Unlike Canada’s commercially managed forests that have a long history of organized inventory and modelling tools [28], there is a lack of coordinated data and considerable uncertainty on assessment procedures for urban forest carbon. To improve upon Canada’s reporting on carbon storage and sequestration by urban forests, this study integrates data collected for Canadian municipalities into the i-Tree Eco model for estimating carbon storage and sequestration for Canada’s urban forests. While the Pasher et al. [22] approach was a starting point for urban forest carbon estimation and reporting in Canada’s national GHG inventory, it does not capture variability in storage and sequestration rates that are observed across Canada’s diverse ecozones and jurisdictions. The goal of this study is to build upon the work already completed by Pasher et al. [22] but integrate data from 16 Canadian urban forest i-Tree Eco assessments conducted across the country. Data from these assessment results were used to develop new carbon storage densities (i.e., carbon per unit area of canopy cover) and sequestration rates at the ecozone level and to re-assess urban forest carbon in Canada. The overarching objective of this study is to update the urban forest portion of the Settlement Category of the land use, land-use change, and forestry (LULUCF) Sector in the national GHG inventory. Continuous improvement in carbon assessment and reporting is a cornerstone of effective mitigation strategies for nations.

## Methods

### Study area

This study integrates several spatial levels of analysis for its national urban forest carbon assessment. The first is the designation of individual urban areas (i.e., part of the Settlement category of the LULUCF sector), which were identified using a Statistics Canada dataset on population centres [25]. This approach was consistent with the study done by Pasher et al. [22]. Population centres greater than 30,000 inhabitants and > 400 people per km^2^ were selected to define urban areas. A total of 86 population centres, which at the time of data collection comprised approximately 67% of the country’s urban area and 76% of the population, were included in the assessment [22]. A spatial framework based on the intersection of Ecozones of Canada and provincial/territorial boundaries were used to define consistent areas used to compile national estimates of urban tree cover. This spatial breakdown yields a total of 60 consistent spatial units from Canada’s 13 provinces/territories and 15 ecozones (18 ecozones after the Boreal Shield, Taiga Shield, and Prairies ecozones are divided into their respective sub-units), referred to as Reconciliation Units (RUs).

**Fig. 1:**
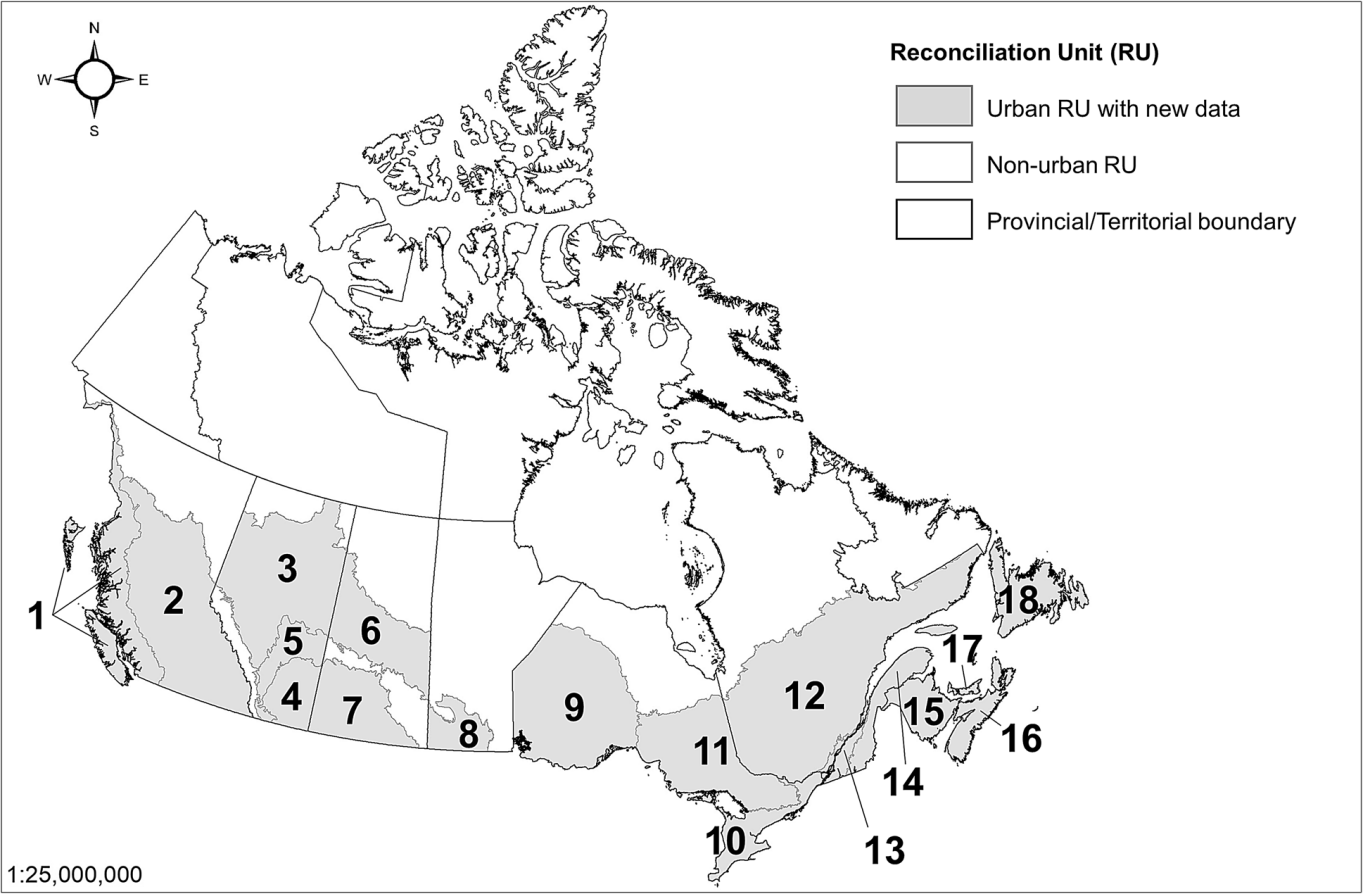
RUs with available urban forest assessment datasets for re-calculating carbon storage and sequestration densities. RU numbering is defined in Table 1

Of these 60 RUs, only 18 contain urban area, as defined above, and are included in the assessment (Fig. 1). Urban forest canopy cover was assessed at the RU level by Pasher et al. [22] and carbon storage and sequestration are assessed and reported at the RU and national level in this study. However, urban forest carbon storage densities and gross sequestration rates were calculated at the ecozone level under the assumption that urban forest structure would be comparable across provincial/territorial boundaries but not ecozone boundaries. While this is a potential limitation of the study as different jurisdictional policies and practices can indeed influence urban forest structure [27], this assumption was made to develop carbon densities for a greater number of RUs and was still seen as an improvement over the current assessment approach of a single storage density and sequestration density for all 18 RUs.

### Urban forest canopy cover

Pasher et al. [22] used a point-sampling approach to determine percent canopy cover for urban areas in the 18 RUs. Canopy cover values were then used to calculate carbon storage and sequestration densities as a function of carbon per unit area of canopy cover. This approach was chosen over a manual classification or unsupervised classification of satellite imagery for being less time intensive while also remaining reliable, given that the aim was to re-assess canopy cover on a regular basis for the national GHG inventory. Moreover, point-based sampling affords the ability to use any imagery, whether it be air photos or multi-spectral satellite imagery. Air photos were the most commonly used imagery and had resolutions ranging from 10 to 30 cm; see Pasher et al. [22] for details.

To obtain canopy cover percentage (Table 1) using the point-sampling method, a 1-km^2^ resolution grid was overlaid on recent aerial imagery for urban areas. A 25% sampling rate of grid cells was used, and each selected grid cell contained 55 evenly spaced points that were classified by landcover type by interpreters. Through iterative testing, an optimal density of 55 points per km^2^ was determined. In conducting the point-sampling canopy cover estimates across the country, several quality control checks were used, as described by Pasher et al. [22]. Any given 1-km^2^ grid cell with 25% or less of its area considered urban was discarded. Any points overlapping with clouds or any other distortions on the imagery were removed. Additional interpreters conducted random checks of point classifications conducted by the first interpreter. In total, 220,000 points were analysed across 4075 km^2^ of urban area with 2500 points removed for errors. This study uses the urban area and canopy cover results from Pasher et al. [22] in its carbon assessment.

**Table 1:** Reconciliation Units (RUs) in Canada and their urban area (i.e., population centres with more that 30,000 people and 400 people per km^2^) and canopy cover in 2011, as described by Pasher et al. [22]

RU	RU (km^2^)	Urban area within RU (km^2^)	Proportion of national urban area (%)	Canopy area (km^2^)	Canopy cover (%)
1. BC Pacific maritime	200,716	1991	12.2	766	38.5
2. BC Montane cordillera	428,955	526	3.2	136	25.8
3. AB Boreal plains	367,539	111	0.7	32	29.0
4. AB Semiarid prairies	71,203	262	1.6	17	6.6
5. AB Subhumid prairies	80,577	1691	10.4	197	11.6
6. SK Boreal plains	164,464	70	0.4	22	32.1
7. SK Semiarid prairies	154,361	340	2.1	40	11.7
8. MB Subhumid prairies	64,079	539	3.3	85	15.8
9. ON Boreal shield west	335,151	183	1.1	87	47.7
10. ON Mixedwood plains	82,439	5317	32.6	1193	22.4
11. ON Boreal shield east	241,097	574	3.5	232	40.3
12. QC Boreal shield east	600,491	142	0.9	47	32.8
13. QC Mixedwood plains	27,707	3240	19.9	961	29.7
14. QC Atlantic maritime	67,077	217	1.3	85	39.0
15. NB Atlantic maritime	71,389	512	3.1	252	49.2
16. NS Atlantic maritime	53,247	337	2.1	168	50.0
17. PE Atlantic maritime	5654	70	0.4	16	22.3
18. NL Boreal shield east	104,740	190	1.2	83	43.8
Total, Canada	3,120,886	16,314	100	4412	27.0

### Urban forest carbon storage and sequestration

The carbon assessment conducted in this study was built upon methods used by Nowak et al. [19] and Pasher et al. [22]. The Nowak et al. [19] study and national assessment is closely linked to both the i-Tree Eco model and the USDA Forest Inventory and Analysis program. i-Tree Eco data were available for 28 US cities in the most recent national assessment. The i-Tree Eco model estimates aboveground and below-ground biomass/carbon stocks and growth/sequestration rates from plot-based field data. Oven-dried biomass by tree species is calculated using allometric equations from previous research to determine carbon storage in individual trees [18]. Where no species-based equations are available, an average of the biomass determined for all species within the genus is used. If genus equations are unavailable, the average is based on either conifer or broadleaf species groupings. Crown light exposure is recorded for every measured tree, which is used to distinguish open-grown trees from forest-grown (i.e., closed canopy conditions) trees. Biomass values of open-grown, maintained trees were multiplied by a factor of 0.8 to account for their lower biomass in comparison to forest-grown trees of the same diameter [18]. Urban tree diameter growth rates estimated by the i-Tree model, and thus sequestration rates, were derived from previous studies for street tree growth rates, park tree growth rates, and forest tree growth rates [18]. The growing season length of each Canadian city where data were collected, crown light exposure, and tree crown condition are used to adjust growth rates further according to local conditions. Lastly, tree biomass was multiplied by 0.5 to attain carbon values Nowak et al. [18].

Pasher et al. [22] conducted a similar assessment of urban forest carbon in Canada at the RU level to inform Canada’s national GHG inventory. The assessment follows the IPCC Tier 2 approach using the following equation:

1 $$\triangle CG = AT \times CRW$$

Where *ΔCG* is the annual carbon accumulation attributed to the biomass increment (t C year^−1^), *AT* is the area of urban canopy cover (ha), and *CRW* is the gross or net sequestration rate for urban trees per unit area of crown cover (t C ha^−1^ year^−1^). In the most recent Canadian GHG inventory [5], *AT* is derived from the point-based urban forest canopy assessment and *CRW* is a fixed gross carbon sequestration rate of 2.12 t C ha^−1^ year^−1^ for the entire country. This rate was based on the gross carbon sequestration rate of 2.77 t C ha^−1^ year^−1^ from the US assessment [19] and adjusted for the average Canadian growing season length of 133 days [22], assuming a linear relationship between growth and growing season length. Similarly, to assess stored carbon stocks in urban forests using the same equation, a fixed carbon density of 76.9 t C ha^-1^ was used for *CRW*, which again was derived from Nowak et al. [19]. The 2.12 t C ha^−1^ year^−1^ rate represents gross carbon sequestration; the net carbon sequestration rate that accounts for carbon emissions from biomass decay is estimated to be 74% of gross rates [19, 22]. Tree mortality effects on carbon sequestration rates are not captured and are a source of uncertainty in estimates; remeasurement of canopy cover and urban areas will capture large mortality events in future national assessments. Carbon storage and sequestration values were converted to CO_2_ by multiplying by the ratio of their atomic weights (i.e., 44/12).

This study addresses two potential limitations of the Pasher et al. [22]. The first is the use of a fixed carbon storage density and sequestration rate for all 18 RUs (and ecozones) with population centres across the country and the second is the fact that these rates are not based on local data, given that Canadian cities are increasingly conducting urban forest assessments and developing management and monitoring plans [21, 27]. There are 16 i-Tree Eco assessments with available data and sufficient sample size for Canadian cities (Table 2). Each dataset includes the assessment area (ha), number of plots, number of measured trees, total carbon storage, and gross carbon sequestration (Table 3). The data were largely collected by municipalities with occasional partnerships with non-governmental organizations (NGOs) and academic institutions. Because no local data were available for the Pacific Maritime ecozone, the City of Seattle’s urban forest assessment data were used as a surrogate. This was done because there is a large urban area in the ecozone with carbon densities that are likely to differ substantially due to the higher productivity of the temperate rainforest ecosystems. Similarly, data from the Subhumid Prairies ecozone were used to develop carbon values for the Semiarid Prairies ecozone and values from Kurz et al. [10] were used for the boreal RUs, as opposed to using the Canada-wide values from Pasher et al. [22].

**Table 2:** Canadian ecozones with population centres of greater than 30,000 people and the communities within that have i-Tree Eco projects with available data that were used in this study

Forested ecozones	Land area (ha)	Urban area (ha)	i-Tree eco data
Atlantic maritime (NB, NS, PE)	19,736,815	113,600	Halifax
Boreal plains^a^ (AB, SK)	67,185,834	18,100	None
Boreal shield east^a^ (NL, ON, QC)	99,129,131	90,600	None
Boreal shield west^a^ (ON)	71,111,613	18,300	None
Mixedwood plains (ON, QC)	11,014,617	855,700	Ajax, Aurora, Bolton, Brampton, Caledon, London, Markham, Mississauga, Oakville, Pickering, Richmond Hill, Toronto
Montane cordillera (BC)	47,226,428	52,600	Kelowna
Pacific maritime^b^ (BC)	20,487,877	199,100	Seattle
Semiarid prairies^c^ (AB, SK)	23,493,794	60,200	None
Subhumid prairies (AB, MB)	21,598,791	250,000	Calgary, Edmonton

^a^Carbon values for Canada’s managed boreal forest from Kurz et al. [10] was used for the Boreal ecozone urban forests

^b^Seattle was used to develop carbon values for the Pacific Maritime ecozone since no Canadian data were available and because it represents a large proportion of the urban area in Canada

^c^The Subhumid Prairies were used to develop carbon rates for the Semiarid Prairies

To calculate new RU carbon values (*CRW*) using the assessment data from Canadian cities, new canopy cover estimates (*AT*) were first calculated for each of the 16 Canadian urban forest assessments using their total assessment area (ha) multiplied by the canopy cover (%) estimates from Pasher et al. [22] for the RU in which the assessment was situated. The City of Seattle had satellite-based canopy cover estimates for the assessment area, which were used to calculate the BC Pacific Maritime carbon densities. Updated carbon density values were then used to estimate total carbon storage, gross sequestration, net sequestration, and CO_2_ estimates at the RU level. Updated assessment estimates were then mapped at the RU level and compared to the Pasher et al. [22] estimates.

**Table 3:** Summary of i-Tree Eco carbon estimates for Canadian urban forest assessments, including total assessment area, number of assessment plots, and estimates of total values and their standard errors (SE)

City	Assessment area (ha)^a^	Plots	Number of trees_b_		Carbon storage (t)		Carbon sequestration (t year^−1^)	
			Total	SE	Mean	SE	Mean	SE
Halifax	69,270	190	57,862,251	4,214,326	2,134,697	156,789	118,483	8228
Ajax	6743	198	1,365,760	228,063	105,641	18,065	3547	471
Aurora	4943	205	1,955,031	265,477	102,981	12,824	4050	418
Bolton	1677	46	311,080	102,217	12,682	4341	757	185
Brampton	26,945	196	3,617,714	776,086	174,736	30,078	7732	1130
Caledon	478	37	302,595	97,972	13,689	3468	590	128
London	23,591	383	4,375,760	479,761	359,763	42,669	12,451	1145
Markham	21,269	213	3,154,950	541,758	229,886	42,226	9229	1413
Mississauga	28,801	205	2,104,091	306,994	202,870	40,786	10,002	1493
Oakville	9893	367	1,849,305	186,485	49,724	4937	4177	390
Pickering	4718	219	1,671,866	194,685	104,191	13,733	4242	428
Richmond hill	10,201	208	2,559,349	322,465	165,699	26,595	7241	876
Toronto	63,727	407	10,220,059	952,441	1,107,645	99,010	46,741	3302
Kelowna	21,723	150	3,302,464	620,998	126,911	19,245	7713	1300
Calgary	55,032	196	2,076,400	498,395	183,595	39,072	10,108	1679
Edmonton	69,985	307	12,808,192	2,106,252	615,774	91,284	31,848	4055
Seattle^c^	54,324	223	1,483,837	–	535,261	–	37,817	–

SE values use the same units as mean values

^a^Assessment area is derived from the study areas delineated during i-Tree Eco assessments and may differ from other defined urban areas for municipalities

^b^Note that standard error values from the i-Tree Eco data are derived from sampling error and not error of estimation, as estimation error is unknown [18]

^c^Seattle data were used to develop carbon rates for the Pacific Maritime ecozone since no Canadian data were available

### Uncertainty assessment

A complete assessment of the i-Tree modelling platform is beyond the scope of this work and has been addressed elsewhere [12]. However based on the empirical data used in the analysis, it was possible to estimate the uncertainty associated with national and regional estimates of carbon storage and sequestration rates. A Monte Carlo analysis was carried out that integrated the various uncertainties that are present in regional activity data (Table 1), i-Tree assessments, and model uncertainty.

The i-Tree assessments provided model runs of carbon sequestration (mean, SE) and storage (mean, SE) from cities with site sampling uncertainty. Estimates of minimum, mode, and maximum on urban area uncertainty were developed assuming a triangular distribution, with a lower boundary (10%) following the approach used in the United States’ 2012 national inventory report [7] for urban areas and upper boundary based on the known area that is currently outside of the analysis, in small urban centres. Tree canopy coverage uncertainty is based on Pasher et al. [22]; Table 1 at 0.2%. Due to the lack of uncertainty assessment in model parameters, the recommended model uncertainty of 20% for the i-Tree model [30] was used to reduce the known bias of the model.

The Monte Carlo analysis was performed in Analytica [1] and structured around three distinct classes of urban area with increasing levels of uncertainty. The first was urban area for cities whose uncertainty ranges were directly provided by the i-Tree model output. The majority of these cities were located within RU 10, with some coverage in RU 1, 2, 5 and 16 (Table 1). The second class was the remaining fraction of urban area within each of these RUs, outside of city areas, and was estimated using the mean of cities plus inter-city variability observed in RU 10. The final class was defined by having no city area from the i-Tree studies, and combined the mean of a representative proxy city with inter-city variability from RU 17 and variability between all RUs. Each of these types of RUs were assigned different rates of uncertainty. As noted, cities with an i-Tree analysis used those data to calculate uncertainty and were then weighted by the fraction of urban area in the city to the total urban area in the RU. Secondly, for cities within RUs with an i-Tree analysis but without city-specific analyses, the distribution was rebuilt using the mean of cities in a given RU, standard deviation (SD) based on inter-city variability seen in the Mixedwood Plains RU, and the weighted fraction of urban area in the RU. Lastly, for RUs with no i-Tree analysis in a contained city, a normal distribution was built by selecting mean and SD values of a representative proxy city plus SD from between cities in the Mixedwood Plains RU and SD from between RUs.

## Results

**Fig. 2:**
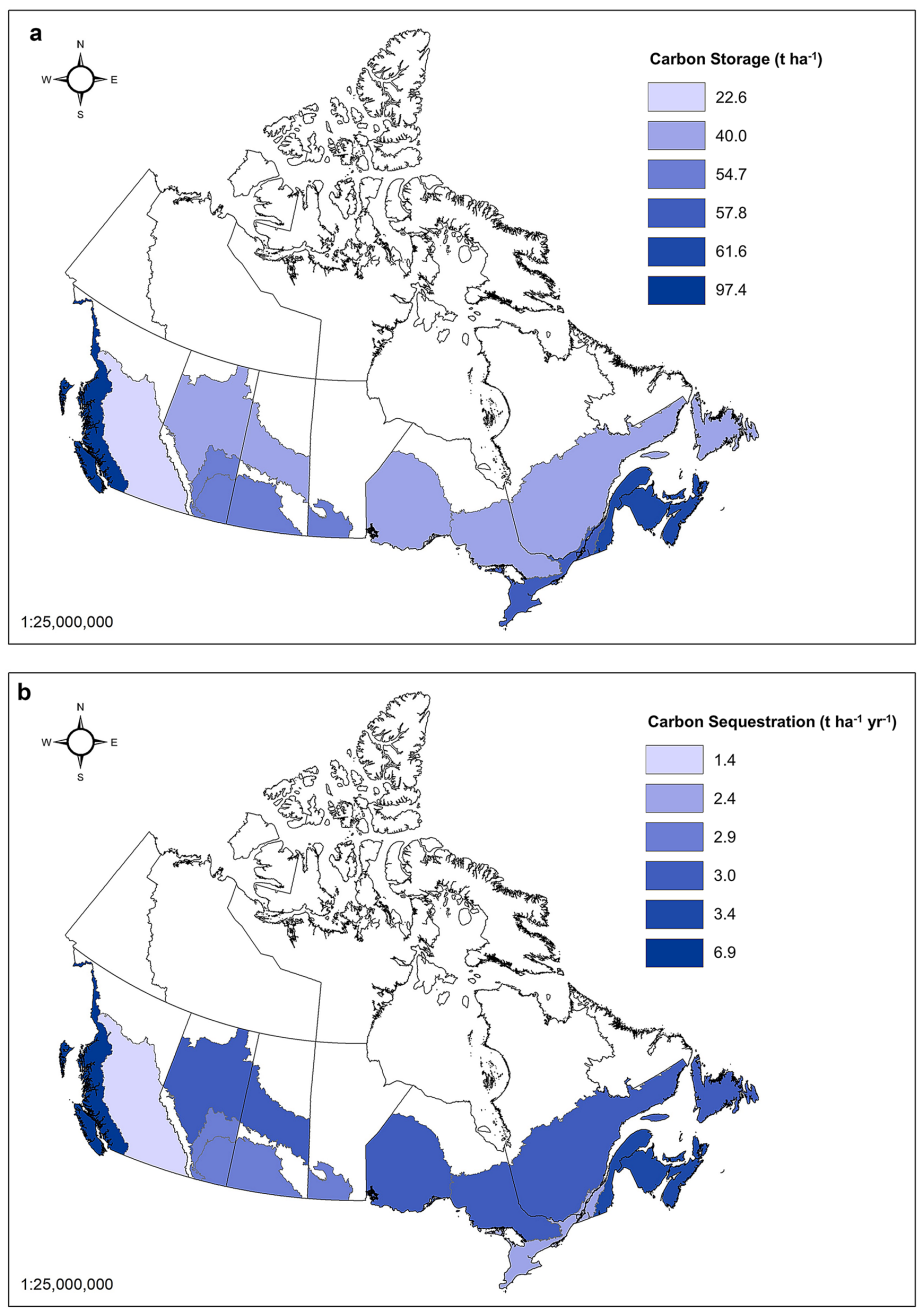
**a** Carbon storage and **b** carbon sequestration densities per unit tree cover for 18 RUs across Canada

Canadian urban forest assessment data allowed for the calculation of updated carbon estimates for 18 RUs within the nine ecozones with urban areas (Table 4). The Mixedwood Plains ecozone (with RUs in ON and QC) and Subhumid Prairies ecozone (with RUs in AB and SK) were the only ecozones with multiple cities having carbon assessment data. Cities in the Mixedwood Plains ecozone had carbon values ranging from 33.8 t ha^−1^ to 127.9 t ha^−1^ for storage and from 1.3 t ha^−1^ year^−1^to 4.0 t ha^−1^ year^−1^ for sequestration. The new carbon storage densities (Table 5; Fig. 2) tended to be lower than the values from Nowak et al. [19] and Pasher et al. [22], while the carbon sequestration densities tended to be higher. Given the architecture of the i-Tree Eco model and its approach to simulating carbon sequestration, these higher values compared to the US must be attributed to faster-growing tree species and smaller trees, better average tree condition, and/or more open-grown trees, since Canada’s shorter growing season would lower sequestration rates with all else being held equal. However, this requires further empirical research. The uncertainty on CO_2_ removals by urban trees lies within a range of − 33% to + 38%, and is the combined uncertainty associated with estimates of urban area, canopy cover, and the C sequestration rate. (Table 5).

**Table 4:** Carbon storage densities and gross sequestration rates per unit of tree cover for Canadian Ecozones

Ecozone	Carbon storage (t ha^−1^)	Carbon sequestration (t ha^− 1^ year^− 1^)	Comments
Pacific maritime	97.4	6.9	Derived from the Seattle [3]
Montane cordillera	22.6	1.4	
Boreal plains	40.0	3.0	Derived from forest models [10]
Subhumid prairies	54.7	2.9	
Semiarid prairies	54.7	2.9	Derived from Subhumid Prairies
Boreal shield west	40.0	3.0	Derived from forest models [10]
Mixedwood plains	57.8	2.4	
Boreal shield east	40.0	3.0	Derived from forest models [10]
Atlantic maritime	61.6	3.4	

**Table 5:** Updated urban forest carbon storage and sequestration by reconciliation unit (RU) for Canada’s urban areas with associated uncertainties

RU	Total carbon storage (kt)	2.5% /97.5% Prob.	Gross carbon sequestration (kt C year^− 1^)	2.5% /97.5% Prob.	Net carbon sequestration (kt C year^− 1^)	2.5% /97.5% Prob.	Net CO_2_ sequestration (kt CO_2_ year^− 1^)	2.5% /97.5% Prob.
1. BC pacific maritime^a^	7456.20	491 (− 93%) / 17,637 (+ 137%)	526.7	61 (− 88%) / 1095 (+ 108%)	395.1	45 (− 88%) / 821 (+ 108%)	1448.50	167 (− 88%) / 3010 (+ 108%)
2. BC Montane cordillera	307.5	89 (− 71%) / 698 (+ 127%)	18.7	13 (− 30%) / 26 (+ 39%)	14	10 (− 30%) / 19 (+ 39%)	51.4	36 (− 30%) / 72 (+ 39%)
3. AB Boreal plains^b^	128.8	9 (− 93%) / 308 (+ 139%)	9.7	1 (− 93%) / 24 (+ 145%)	7.2	0 (− 93%) / 18 (+ 145%)	26.4	2 (− 93%) / 65 (+ 145%)
4. AB Semiarid prairies^c^	94.6	6 (− 94%) / 232 (+ 145%)	5	0 (− 93%) / 12 (+ 132%)	3.7	0 (− 93%) / 9 (+ 132%)	13.7	1 (− 93%) / 32 (+ 132%)
5. AB Subhumid prairies	1076.60	736 (− 32%) / 1573 (+ 46%)	56.5	46 (− 18%) / 68 (+ 21%)	42.4	35 (− 18%) / 51 (+ 21%)	155.4	127 (− 18%) / 188 (+ 21%)
6. SK Boreal plains^b^	89.6	6 (− 93%) / 213 (+ 137%)	6.8	0 (− 93%) / 17 (+ 145%)	5	0 (− 93%) / 12 (+ 145%)	18.4	1 (− 93%) / 45 (+ 145%)
7. SK Semiarid prairies^c^	216.4	13 (− 94%) / 526 (+ 143%)	11.4	1 (− 93%) / 26 (+ 130%)	8.5	1 (− 93%) / 20 (+ 130%)	31.2	2 (− 93%) / 72 (+ 130%)
8. MB Subhumid prairies^c^	466.7	28 (− 94%) / 1132 (+ 143%)	24.5	2 (− 93%) / 57 (+ 131%)	18.4	1 (− 93%) / 42 (+ 131%)	67.4	5 (− 93%) / 156 (+ 131%)
9. ON Boreal shield west^b^	349.2	18 (− 95%) / 917 (+ 163%)	26.4	1 (− 95%) / 66 (+ 150%)	19.5	1 (− 95%) / 49 (+ 150%)	71.5	4 (− 95%) / 179 (+ 150%)
10. ON Mixedwood plains	6901.50	2892 (− 58%) / 12,086 (+ 75%)	290.7	252 (− 13%) / 337 (+ 16%)	218	189 (− 13%) / 253 (+ 16%)	799.4	693 (− 13%) / 928 (+ 16%)
11. ON Boreal shield east^b^	926.4	91 (− 90%) / 2037 (+ 120%)	69.9	7 (− 90%) / 154 (+ 120%)	51.8	5 (− 90%) / 114 (+ 120%)	189.8	19 (− 90%) / 417 (+ 120%)
12. QC Boreal shield east^b^	186.4	18 (− 90%) / 422 (+ 127%)	14.1	1 (− 91%) / 32 (+ 125%)	10.4	1 (− 91%) / 23 (+ 125%)	38.2	4 (− 91%) / 86 (+ 125%)
13. QC Mixedwood plains	5557.00	411 (− 93%) / 12,860 (+ 131%)	234.1	19 (− 92%) / 532 (+ 127%)	175.6	14 (− 92%) / 399 (+ 127%)	643.7	52 (− 92%) / 1463 (+ 127%)
14. QC Atlantic maritime	522	36 (− 93%) / 1259 (+ 141%)	29	3 (− 91%) / 66 (+ 127%)	21.7	2 (− 91%) / 49 (+ 127%)	79.7	7 (− 91%) / 181 (+ 127%)
15. NB Atlantic maritime	1552.00	105 (− 93%) / 3743 (+ 141%)	86.1	8 (− 91%) / 193 (+ 124%)	64.6	6 (− 91%) / 145 (+ 124%)	236.9	22 (− 91%) / 530 (+ 124%)
16. NS Atlantic maritime	1037.30	804 (− 22%) / 1359 (+ 31%)	57.6	45 (− 22%) / 75 (+ 31%)	43.2	34 (− 22%) / 57 (+ 31%)	158.3	123 (− 22%) / 207 (+ 31%)
17. PE ATlantic maritime	96.8	7 (− 93%) / 229 (+ 136%)	5.4	1 (− 90%) / 12 (+ 119%)	4	0 (− 90%) / 9 (+ 119%)	14.8	1 (− 90%) / 32 (+ 119%)
18. NL Boreal shield east^b^	332.8	31 (− 91%) / 738 (+ 122%)	25.1	2 (− 91%) / 56 (+ 122%)	18.6	2 (− 91%) / 41 (+ 122%)	68.2	6 (− 91%) / 151 (+ 122%)
Total, Canada	27,297.80	17,257 (− 37%) / 39,347 (+ 44%)	1497.70	1008 (− 33%) / 2063 (+ 38%)	1121.70	755 (− 33%) / 1545 (+ 38%)	4112.90	2767 (− 33%) / 5666 (+ 38%)

^a^Carbon values derived from the Seattle, WA, i-Tree Eco report [3] were used for the BC Pacific Maritime RU

^b^Carbon values for Canada’s managed boreal forest from Kurz et al. [10] was used for the Boreal RUs

^c^Carbon values from AB Subhumid Prairies (i.e., Edmonton and Calgary assessments) were used for AB Semiarid, SK Semiarid, and MB Subhumid RUs

**Fig. 3:**
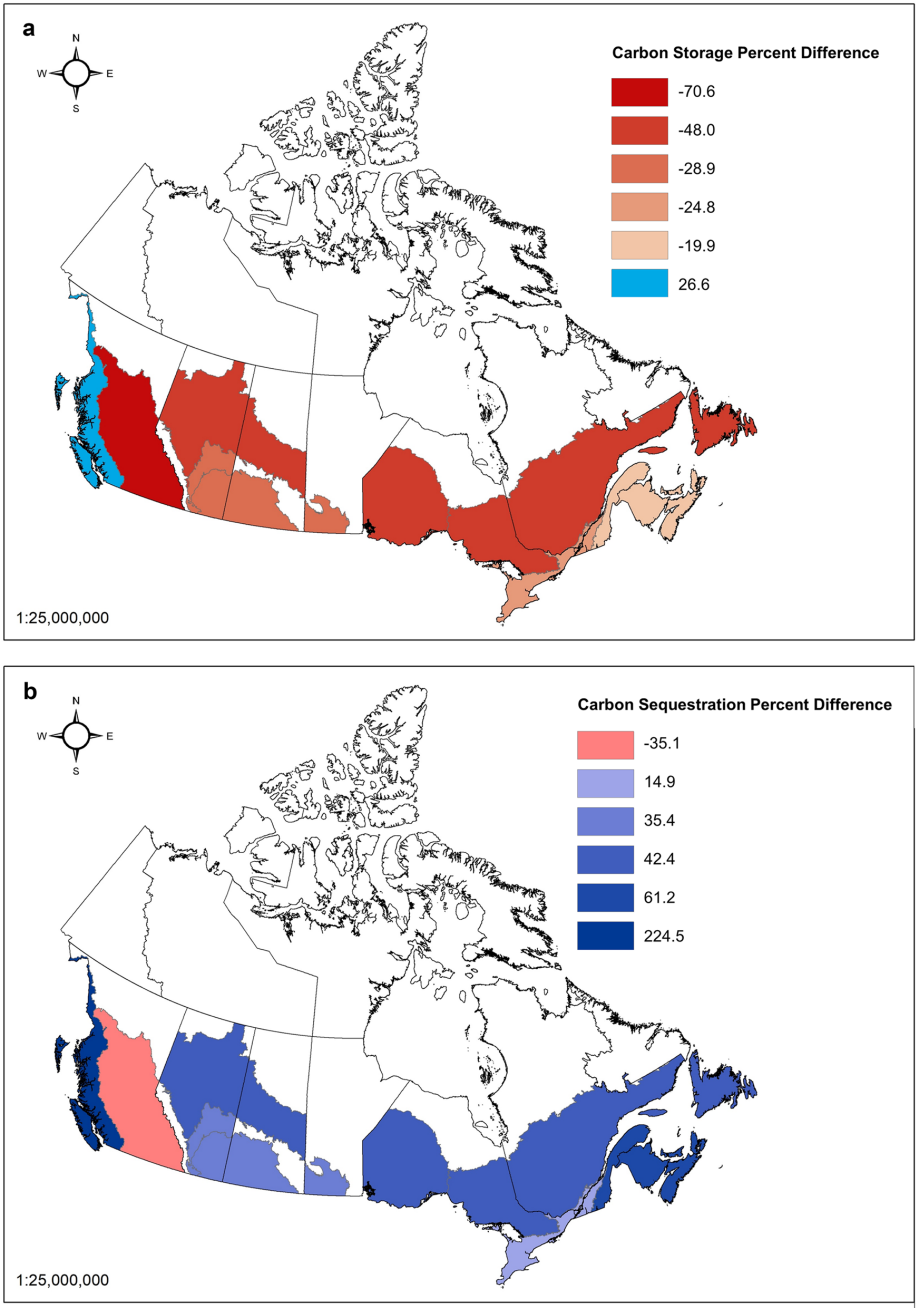
A comparison of **a** carbon storage and **b** carbon sequestration assessed in this study with the assessed carbon values presented in Pasher et al. [22]

The results of this analysis confirm the presence of variability in urban forest carbon dynamics across RUs and ecozones in Canada (Table 5; Fig. 2). Carbon storage on average is equivalent to 46 t C ha^−1^ (-37%, + 44%) with observed rates varying from 97 to 23 kt C ha^−1^ and sequestration rates were on average 3.2 t C ha^−1^ year^−1^ (− 33%, + 38%) varying from a minimum of 1.4 to 6.9 t C ha^−1^ year^−^1. The lowest levels of carbon storage and sequestration were found in the BC Montane Cordillera RU based on the Kelowna urban forest assessment. The BC Pacific Maritime RU had the highest rates of carbon storage and sequestration, based on the urban forest assessment in Seattle. When comparing the results to those of Pasher et al. [22], carbon storage densities based on Canadian data were consistently lower than the extrapolation of US data that was used in that study, with the exception of BC Pacific Maritime and sequestration rates were consistently higher with the exception of BC Montane Cordillera (Table 6). Total carbon storage in Canada was estimated to be 6,684.6 kt C ha^−1^ lower than Pasher et al. [22], while gross sequestration was found to be 560.8 kt C year^−1^ higher (Fig. 3).

**Table 6:** Difference between assessed carbon values and assessed carbon values from Pasher et al. [22]

Reconciliation units	Total carbon storage (kt)	Total carbon sequestration (kt year^− 1^)	Net carbon sequestration (kt year^− 1^)	Net CO_2_ sequestration (kt year^− 1^)
1. BC Pacific maritime_1_	1568.7	364.4	275.0	1008.1
2. BC Montane cordillera	− 737.0	− 10.1	− 7.3	− 26.7
3. AB Boreal plains_2_	− 118.5	2.9	2.2	7.9
4. AB Semiarid prairies_3_	− 38.6	1.3	1.0	3.7
5. AB Subhumid prairies	− 438.4	14.7	11.5	42.1
6. SK Boreal plains_2_	− 83.0	2.0	1.4	5.5
7. SK Semiarid prairies_3_	− 88.4	3.0	2.3	8.4
8. MB Subhumid prairies_3_	− 190.0	6.4	5.0	18.3
9. ON Boreal shield west_2_	− 322.4	7.9	5.8	21.3
10. ON Mixedwood plains	− 2276.1	37.7	30.8	112.9
11. ON Boreal shield east_2_	− 854.3	20.8	15.5	56.6
12. QC Boreal shield east_2_	− 171.7	4.2	3.1	11.4
13. QC Mixedwood plains	− 1832.6	30.4	24.8	90.9
14. QC Atlantic maritime	− 129.3	11.0	8.4	31.0
15. NB Atlantic maritime	− 384.6	32.7	25.1	92.0
16. NS Atlantic maritime	− 257.2	21.9	16.8	61.5
17. PE Atlantic maritime	− 23.9	2.1	1.5	5.8
18. NL Boreal shield east_2_	− 307.3	7.5	5.5	20.3
Total, Canada	− 6684.6	560.8	428.3	1571.0

Negative values indicate results lower than those found in Pasher et al. [22]

## Discussion

Urban forests play an important role in the carbon cycle of cities and are the primary source of atmospheric carbon removals within urban land uses [16]. Using canopy cover estimates derived from ortho-imagery and satellite imagery ranging from 2008 to 2012 [22] and i-Tree Eco urban forest assessment data from 16 Canadian cities and one US city, this study found that Canadian urban forests store approximately 27,297.8 kt C (− 37%, + 45%) and sequester (gross) approximately 1497.7 kt C year^−1^ (− 26%, + 28%). Urban forests help to reduce emissions indirectly in several other sectors, such as reducing energy-sector emissions by reducing urban heat island effects and shading buildings [4]).

This analysis provides an important cross-validation of the Pasher et al. [22] work. Differences in urban forest structure largely drive differences in carbon storage and sequestration, across Canadian ecozones. The Pasher et al. [22] analysis used a single carbon storage and sequestration value provided by Nowak et al. [19], with the sequestration rate being adjusted to a lower rate to reflect a shorter but uniform growing season in Canada compared to the US. While a correction factor was developed to consider differences in climate, the analysis did not necessarily consider differences in the urban forest structure and the relative weight that differences in urban forests could have on the national total. Carbon densities provided an important starting point for understanding urban forest carbon at the national level and for improving estimates and methodologies for LULUCF carbon accounting in the Settlements category of the national GHG inventory. The rapid proliferation of urban forest plans and strategies in Canadian urban communities has translated into a greater availability of local urban forest data and the capacity to refine these estimates and capture cross-country variability. Importantly, the definition of what qualifies as an urban area has considerable implications for total urban forest carbon balances in Canada and would vary if population size or density constraints were adjusted.

The Pasher et al. [22] study suggest that carbon storage in urban trees across the country may have been overestimated, with the exception of the BC Pacific Maritime RU. As this RU contains the Coast Forest Region, which is a temperate rainforest experiencing longer growing seasons with higher rates of precipitation and much larger trees per unit area of canopy cover, it is expected that carbon values would be much higher than the base storage rate for the country [6, 23]. Conversely, the Prairies ecozones (i.e., AB Semihumid Prairies, AB Semiarid Prairies, SK Semiarid Prairies, and MB Subhumid Prairies RUs) and Montane Cordillera ecozone/BC Montane Cordillera RU have much lower carbon storage and sequestration rates, likely due to their drier climate, open woodland/prairie conditions [6, 8], and resulting slower growth rates of urban trees under these conditions. These conditions can arguably translate to fewer trees and/or trees with lower biomass per unit area of canopy cover, though this needs further empirical testing. Moreover, in such settings urban forest canopy cover may be higher than the surrounding natural landscape [17].

At the time of completing this study, there were no suitable carbon data from urban forest estimates in any of the boreal RUs and data from commercially managed boreal forests were used [10]. While these boreal RUs represent less than 8% of the Canadian urban area defined in this study, the RUs represent a large proportion of the country’s total area and contain several large urban centres with populations over 100,000, such as Thunder Bay and Sudbury, ON, Saguenay, QC, and St. John’s, NL, and many more still that meet the urban definition of greater than 30,000 people and 400 people per km^2^.

In stark contrast, carbon sequestration was consistently underestimated in the previous assessment compared to the Nowak et al. [19] US value of 2.77 t C ha^−1^ year^−1^ (standard error = 0.45 t C ha^−1^ year^−1^) and Pasher et al. [22] Canadian value of 2.12 t C ha^−1^ year^−1^ (standard error = 0.45 t C ha^−1^ year^−1^), with the exception of the BC Montane Cordillera RU. The largest sequestration value of 6.9 t C ha^−1^ in the BC Pacific Maritime RU is roughly three times the sequestration rate of the previous assessments, which is likely due to the large trees in this RU and subsequently larger carbon sequestration with a given diameter growth increment. Further, due to the relatively high proportion of urban area in this region, these urban forests have an important impact on the national average and should be studied further as data were derived from Seattle and also lack detailed uncertainty estimates.

Differences in urban forest structure and associated carbon values across RUs and ecozones can likely be attributed to the differing climate, site conditions, and tree species. Another possible, albeit speculative, explanation is a younger urban forest canopy in Canada, which would yield both lower carbon storage and higher sequestration (i.e., growth) rates per unit area of canopy cover. Yet another possible explanation in some urban areas could be a greater abundance of softwood species or lower-density hardwood species (e.g., *Populus* species in the Prairie cities), both of which have lower specific gravities and thus less carbon stored and sequestered in a given tree compared to many common US hardwood species [15].

However, it is also highly likely that some fraction of the differences in carbon density values can be attributed to methodological differences between sources of data, which is an important source of uncertainty in this study. The 16 i-Tree Eco urban forest assessment areas represent small proportions of the urban area in a given RU and may have differing canopy cover values than the total urban canopy cover in a given RU, which would influence the carbon storage and sequestration densities calculated in this study. While the i-Tree Eco urban forest assessments include canopy cover estimates, these estimates are based on plot-level visual assessment by field crews and it was deemed that the point-based canopy cover values from Pasher et al. [22] would be more reliable. Moreover, the latter canopy cover assessment methods will be used to account for urban trees in national GHG inventories.

There are other study limitations worthy of mention. A total of 16 urban centres in Canada had conducted assessments with usable carbon data. While the Mixedwood Plains ecozone had 12 population centres with datasets, the Montane Cordillera and Atlantic Maritime ecozones each had only one population centre. The Montane Cordillera ecozone had lower values for storage and sequestration than is typically observed in urban forests [19]. In other cases, RUs were assigned carbon densities from neighbouring and closely related ecozones, such as the Subhumid and Semiarid Prairies RUs or the BC Pacific Maritime RU. Regardless of sources of error, the present approach assures that as new communities continue to actively manage their urban forests and conduct new local assessments, carbon estimates from this study’s assessment and subsequently the national GHG inventory values can be continually updated and improved upon. Lastly, future research might investigate building upon existing research on Canadian biomass/carbon equations (e.g., [11] by validating existing equations and developing new ones for common urban tree species in urban settings.

The major driver of uncertainty at the RU level was associated with sample coverage. While uncertainty on carbon storage and sequestration rates within cities covered by i-Tree was often low, uncertainty outside of these areas was high based on the use of proxies and variability between cities and RUs. The RU with the highest uncertainty was the ON Boreal Shield West at 150%, where there were multiple cities in the RU without i-Tree assessments and a proxy city in a different RU. The RU with the smallest uncertainty was the ON Mixedwood Plains at 16%, which has the highest proportion of urban area covered by i-Tree assessments. The model uncertainty for carbon sequestration and storage was considered to be 20%, based on the recommendation in the i-Tree model documentation [30]. However, a comparative study of 15 cities in the Untied States found that carbon sequestration and storage in the i-Tree model uncertainty or at least the precision of the model to be much lower at 2.6% and 2.1% respectively than the higher recommendation in the model documentation [12]. The national uncertainty on CO_2_ removals from urban trees in Canada was 33%. When the uncertainty of the urban area and the uncertainty on urban tree canopy cover (0.2%; [22]) were taken into consideration, the national uncertainty around total carbon sequestered for Canada was 38%. Further studies are required that measure the model uncertainty in using the i-Tree model and its parameters.

There are also some key areas for future research and assessment of urban forest carbon. Beyond calculating carbon storage and sequestration in living aboveground and belowground urban forest biomass, future research could address carbon fluxes in urban forest dead organic matter, soils, and harvested wood products. Regarding the latter, urban trees are often chipped and composted after removal, thus leading to relatively rapid release of their stored carbon through decomposition [9]. There exists an opportunity to better utilize urban trees that have reached the end of their functional lifespan in longer-lived, value-added harvested wood products [2]. Lastly, to further improve our understanding of carbon in urban centres, future research might also look at the value of stratifying urban canopy and carbon estimates by land use within a given urban area, given the strong influence of land use on urban forest structure [26]. Essential to all of these areas of future research is public-sector commitment to regular monitoring of urban forests and urban forestry.

Urban forests are vital ecosystem service providers in cities and, despite some associated costs and hazards, largely benefit a city’s biodiversity, infrastructure, and health and wellbeing of urban inhabitants [4]. Maximizing urban forest carbon sinks will contribute to Canada’s mitigation efforts and, while being a smaller carbon sink compared to commercial forests, will also provide these important aforementioned co-benefits to approximately 83% of Canadian people. Moreover, many of the reductions in carbon emissions associated with urban trees (e.g., avoided energy-based emissions from building energy conservation) represent permanent, not temporary, emissions reductions [20]. Continually improving GHG accounting and reporting in the country’s GHG inventory methods is an important responsibility of the federal government for its climate change mitigation efforts. Moreover, the government investment in national GHG inventories can be leveraged to improve the overall quality of urban forest monitoring and enable local communities to better understand and manage their urban forests across Canadian cities. These same cities are also conducting research, monitoring, and generating data at local scales that have high value for federal government monitoring and reporting.

## Data Availability

No additional data or materials for this study are available. Only aggregate i-Tree Eco data and summaries for Canadian and American cities were used for this study and can be seen in the manuscript tables. Raw i-Tree Eco data from the 17 individual cities are not publicly available.

## References

[1] Analytica. (n.d.). Version (4.5). Lumina Decision Systems—Analytics Software—Visual Modeling. https://analytica.com/. Accessed July 07 2023.

[2] Bratkovich S, Bowyer J (2008). Urban tree utilization and why it matters.

[3] Ciecko L, Tenneson K, Dilley J, Wolf K (2012). Seattle’s forest ecosystem values: analysis of the structure, function, and economic benefits.

[4] Duinker P, Ordóñez C, Steenberg J, Miller K, Toni S, Nitoslawski S (2015). Trees in canadian cities: indispensable life form for urban sustainability. Sustainability.

[5] ECCC (2022). National inventory report 1990–2020: greenhouse gas sources and sinks in Canada.

[6] Ecological Stratification Working Group (1995). A national ecological framework for Canada.

[7] U.S. EPA. United States environmental protection agency. Inventories of U.S. greenhouse gas emissions and sinks: 1990–2010. EPA Report 430-R-12-001. 2013.

[8] Hogg EH, Bernier PY (2005). Climate change impacts on drought-prone forests in western Canada. Forestry Chron.

[9] Kurz WA, Apps MJ (1999). A 70-year retrospective analysis of carbon fluxes in the canadian forest sector. Ecol Appl.

[10] Kurz WA, Shaw CH, Boisvenue C, Stinson G, Metsaranta J, Leckie D, Dyk A, Smyth C, Neilson ET (2013). Carbon in Canada’s boreal forest—a synthesis. Environ Rev.

[11] Lambert MC, Ung CH, Raulier F (2005). Canadian national tree aboveground biomass equations. Can J For Res.

[12] Lin J, Krill CN, Nowak DJ (2021). An uncertainty framework for i-tree eco: a comparative study of 15 cities across the United States. Urban For Urban Green..

[13] Loughner CP, Allen DJ, Zhang D, Pickering KE, Dickerson RR, Landry L (2012). Roles of urban tree canopy and buildings in urban heat island effects: parameterization and preliminary results. J Appl Meteorol Climatol.

[14] McGovern M, Pasher J (2016). Canadian urban tree canopy cover and carbon sequestration status and change 1990–2012. Urban For Urban Green.

[15] Miles PD, Smith WB (2009). Specific gravity and other properties of wood and bark for 156 tree species found in North America.

[16] Nowak DJ, Crane DE (2002). Carbon storage and sequestration by urban trees in the USA. Environ Pollut.

[17] Nowak DJ, Greenfield EJ (2010). Evaluating the national land cover database tree canopy and impervious cover estimates across the conterminous United States: a comparison with photo-interpreted estimates. Environ Manage.

[18] Nowak DJ, Crane DE, Stevens JC, Hoehn RE, Walton JT, Bond J (2008). A ground-based method of assessing urban forest structure and ecosystem services. Arboric Urban For.

[19] Nowak DJ, Greenfield EJ, Hoehn RE, Lapoint E (2013). Carbon storage and sequestration by trees in urban and community areas of the United States. Environ Pollut.

[20] Nowak DJ, Appleton N, Ellis A, Greenfield E (2017). Residential building energy conservation and avoided power plant emissions by urban and community trees in the United States. Urban For Urban Green.

[21] Ordóñez C, Duinker PN (2013). An analysis of urban forest master plans in Canada: implications for urban forest management. Landsc Urban Plann.

[22] Pasher J, McGovern M, Khoury M, Duffe J (2014). Assessing carbon storage and sequestration by Canada’s urban forests using high resolution earth observation data. Urban For Urban Green.

[23] Pojar J, Klinka K, Meidinger DV (1987). Biogeoclimatic ecosystem classification in British Columbia. For Ecol Manag.

[24] Salmond J, Tadaki M, Vardoulakis S, Arbuthnott K, Coutts A, Demuzere M, Dirks K, Heaviside C, Lim S, Macintyre H, McInnes R, Wheeler B (2016). Health and climate related ecosystem services provided by street trees in the urban environment. Environ Health.

[25] Statistics Canada. Population centre boundary file, 2011 census year. catalogue No. 92-166-XWE. 2011. https://www12.statcan.gc.ca/census-recensement/2011/geo/bound-limit/bound-limit-2011-eng.cfm. Accessed 07 Jul 2023.

[26] Steenberg JW, Millward AA, Duinker PN, Nowak DJ, Robinson PJ (2015). Neighbourhood-scale urban forest ecosystem classification. J Environ Manage.

[27] Steenberg JW, Duinker PN, Nitoslawski SA (2019). Ecosystem-based management revisited: updating the concepts for urban forests. Landsc Urban Plann.

[28] Stinson G, Kurz WA, Smyth CE, Neilson ET, Dymond CC, Metsaranta JM, Boisvenue C, Rampley GJ, Li Q, White TM, Blain D (2011). An inventory-based analysis of Canada’s managed forest carbon dynamics, 1990 to 2008. Glob Change Biol.

[29] USDA Forest Service. i-tree: tools for assessing and managing forests & community trees. 2019. https://www.itreetools.org/eco/overview.php. Accessed 07 Jul 2023.

[30] USDA Forest Service. i-tree landscape methods, limitations and uncertainties. 2021. https://www.itreetools.org/documents/115/Landscape_Methods.pdf. Accessed 07 Jul 2023.

[31] Zhao M, Kong Z, Escobedo FJ, Gao J (2010). Impacts of urban forests on offsetting carbon emissions from industrial energy use in Hangzhou, China. J Environ Manag.

